# The alteration and role of glycoconjugates in Alzheimer’s disease

**DOI:** 10.3389/fnagi.2024.1398641

**Published:** 2024-06-13

**Authors:** Yue Kang, Qian Zhang, Silu Xu, Yue Yu

**Affiliations:** ^1^College of Pharmacy, Nanjing University of Chinese Medicine, Nanjing, Jiangsu, China; ^2^Department of Pharmacology, School of Medicine, Nanjing University of Chinese Medicine, Nanjing, Jiangsu, China; ^3^Department of Pharmacy, Jiangsu Cancer Hospital & Jiangsu Institute of Cancer Research & The Affiliated Cancer Hospital of Nanjing Medical University, Nanjing, Jiangsu, China; ^4^School of Pharmacy, Fujian Medical University, Fuzhou, Fujian, China

**Keywords:** Alzheimers disease, glycoconjugates, sialyltransferase, fucosyltransferase, glycation

## Abstract

Alzheimer’s disease (AD) is a prevalent neurodegenerative disorder characterized by abnormal protein deposition. With an alarming 30 million people affected worldwide, AD poses a significant public health concern. While inhibiting key enzymes such as *β*-site amyloid precursor protein-cleaving enzyme 1 and *γ*-secretase or enhancing amyloid-*β* clearance, has been considered the reasonable strategy for AD treatment, their efficacy has been compromised by ineffectiveness. Furthermore, our understanding of AD pathogenesis remains incomplete. Normal aging is associated with a decline in glucose uptake in the brain, a process exacerbated in patients with AD, leading to significant impairment of a critical post-translational modification: glycosylation. Glycosylation, a finely regulated mechanism of intracellular secondary protein processing, plays a pivotal role in regulating essential functions such as synaptogenesis, neurogenesis, axon guidance, as well as learning and memory within the central nervous system. Advanced glycomic analysis has unveiled that abnormal glycosylation of key AD-related proteins closely correlates with the onset and progression of the disease. In this context, we aimed to delve into the intricate role and underlying mechanisms of glycosylation in the etiopathology and pathogenesis of AD. By highlighting the potential of targeting glycosylation as a promising and alternative therapeutic avenue for managing AD, we strive to contribute to the advancement of treatment strategies for this debilitating condition.

## 1 Introduction

Alzheimer’s disease (AD) is a devastating neurodegenerative disorder (NDD) characterized by abnormal accumulation of amyloid-β (Aβ) and neurofibrillary tangles resulting from excessive tau phosphorylation. It affects over 30 million individuals worldwide, predominantly seniors, leading to impaired memory, loss of independence, and in severe cases, death (Calsolaro and Edison, 2016). Despite the “amyloid cascade hypothesis” being widely accepted as the primary pathogenic mechanism of AD for decades, traditional anti-amyloid strategies have yielded disappointing results (Khan et al., 2020). Hence, there is an urgent need for novel disease-modifying treatments (Winblad et al., 2016).

Protein glycosylation is among the most abundant and complex types of post-translational modifications in eukaryotes (Ceroni et al., 2008). Glycoconjugates, which are formed by covalent bonds between sugars, proteins, lipids, and other molecules including glycoproteins, proteoglycans, and glycolipids are widely distributed in mammalian cells. Glycans, including *N-*glycans and *O-*glycans, are carbohydrate sequences attached to the ends of proteins or lipids; they are present in the cells of all living organisms and represent the most structurally diverse class of molecules in nature (Chung et al., 2017). Glycans are highly susceptible to diverse pathophysiological environments, and altered glycosylation can regulate signal transduction, thereby influencing disease pathogenesis (Mereiter et al., 2019). For instance, comprehensive glycomics analyses in rats and humans have revealed that significant fucosylation of *N-*glycans is strongly associated with the development of hepatocellular carcinoma, colorectal cancer, and lung cancer (Gao et al., 2015). Moreover, distinct alterations in sialylated and fucosylated *N-* and *O-*glycans in serum proteins have been identified as potential biomarkers for patients with cancer and healthy individuals (Gao et al., 2015). Besides, RNAs can also be included in the world of glycans (Chevet et al., 2021; Flynn et al., 2021; Li et al., 2023). For instance, Flynn et al. (2021) found that glycans exist on the surface RNA of cell membranes; some non-coding RNAs are glycosylated (called glycan-modified RNAs or glycoRNAs) and expressed on the surface of the plasma membrane. The authors proposed that glycoRNAs are potential ligands for the members of the Siglec receptor family and may be involved in the pathophysiological processes, such as serving as triggering factors and/or targets for autoantibodies in autoimmune diseases like systemic lupus erythematosus, where anti-RNA antibodies have been reported previously. Furthermore, the dysregulation of glycoRNAs in a disease may change the RNA functions. A recent study proposed a solid-phase chemoenzymatic method (SPCgRNA) for targeting glycosylated RNAs and found that miRNA, small nucleolar RNA (snoRNA), small nuclear RNA (snRNA), rRNA, and Y_RNA are all glycosylated RNA substrates (Li et al., 2023). The authors also demonstrated differential *N*-glycosylation of small RNAs in hTERT-HPNE and MIA PaCa-2 cancer cells using SPCgRNA. They found that differential miRNA glycosylation affected tumor cell proliferation and survival. Considering the importance of protein glycosylation and RNA glycosylation in biological processes and functions, monitoring altered glycosylation is crucial for disease prevention, diagnosis, and treatment.

In the human CNS, widely distributed *N-*glycans, *O-*glycans, and gangliosides (GGs) contribute to highly efficient learning, memory maintenance, and neurodevelopment (Iqbal et al., 2019). As key mediators of cellular interaction, communication, molecular trafficking, and differentiation (Bukke et al., 2020), abnormal protein glycosylation levels have been closely associated with the development of diabetes, cancer, and several NDDs, including AD (Hung et al., 1980; Videira and Castro-Caldas, 2018; Conroy et al., 2021). This review summarizes current findings on alterations in glycosylation and the molecular mechanisms involved in AD progression, aiming to enhance our understanding of AD pathogenesis and identify new therapeutic strategies for addressing this challenging condition.

## 2 Glycoconjugates in CNS

**Figure 1:**
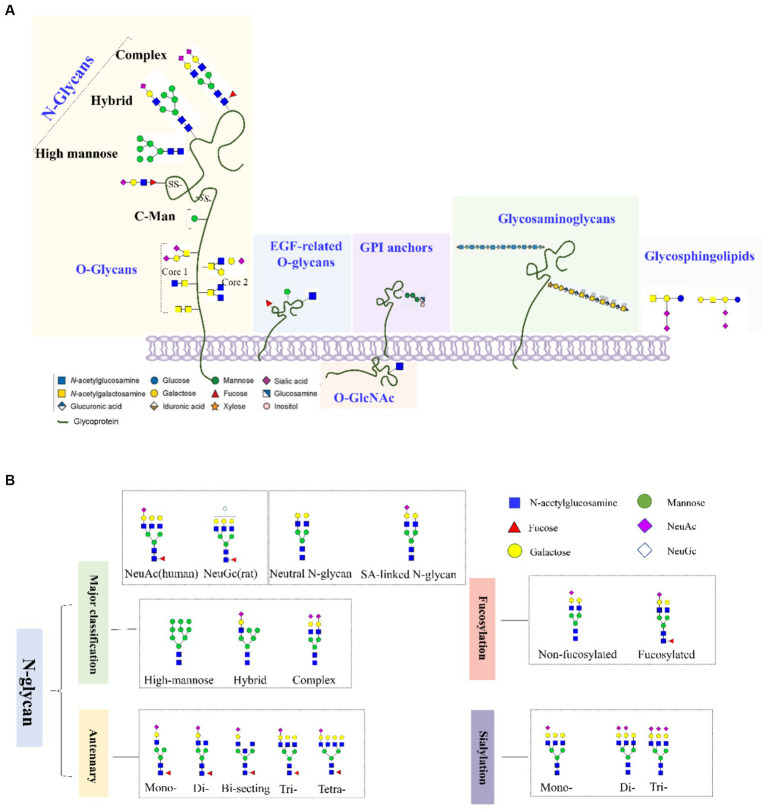
**(A)** Common types of glycoconjugates in human cells and **(B)** *N-*glycans are classified based on high mannose/hybrid/complex structure, the numbers of antennary, fucose, and SAs.

Glycosylation involves the covalent attachment of glycans to the polypeptide backbone, including the formation of *N-*glycans by *N-*linking to asparagine or *O-*glycans by *O-*linking to serine or threonine (Figures 1A,B). *N-*glycosylation involves the covalent attachment of various branched sugars in the endoplasmic reticulum and Golgi, while *O-*glycosylation involves the initial attachment of several monosaccharides like *N-*acetylgalactosamine (GALNAc), mannose, fucose, and galactose (GAL) (Gao et al., 2015). Another type of *O-*glycosylation is *O-*GlcNAcylation in the cytoplasm, involving the attachment of GALNAc to serine or threonine residues (Nie and Yi, 2019; Zhu and Hart, 2021). In the CNS, various glycoconjugates, including *N-*glycans, *O-*glycans, glycosaminoglycans, hyaluronic acid, glycosphingolipids, glycosylphosphatidylinositol anchors, and *O-*GlcNAc, are enriched. The CNS is one of the tissues with the highest lipid content and complexity in mammals (Svennerholm and Raal, 1956).

One of the most abundantly distributed glycoconjugates in the CNS are glycosphingolipids, which are composed of sphingosine connecting a fatty acid chain and a hydrophilic monosaccharide or oligosaccharide and can be mainly classified as GM1, GD1a, GD1b, and GT1b (Klenk, 1942; Garcia-Ruiz et al., 2015). GGs, which are sialic acid-containing glycosphingolipids, play important roles in synaptic transmission, energy supply, cell–cell interactions, and neuronal differentiation. They are crucial for maintaining normal adult brain development (Schengrund, 1990; Haughey, 2010). For example, GD3, a key player in cellular signaling in the CNS, is highly expressed in neural stem cells and can serve as a biomarker for targeting these cells (Nakatani et al., 2010). Moreover, GM1 and GD1a are enriched on the neuronal membrane and play roles in maintaining neuronal function and transmitting signaling (Iqbal et al., 2019). Since GGs are highly expressed in the CNS, the brain is one of the most affected organs by lysosomal storage disorders, as it causes neurons to become distended and eventually die (Sandhoff and Harzer, 2013). This particular accumulation can trigger the onset of several NDDs, such as AD (Yanagisawa, 2007; Ariga et al., 2008; Oikawa et al., 2009).

As the brain develops, the GG expression and patterns change (Robert et al., 2009, 2011; Kolter, 2012). It was found that the composition and quantity of GGs in the brain change with age (Kolter, 2012). During brain growth and development, complex glycosphingolipids, such as GD1a and GT1b, dominate adult brains, while GD2 and GD3, which have simpler structures, predominate the embryonic brains (Nakatani et al., 2010). Sarbu et al. (2017) used Orbitrap MS optimized in the negative ion mode to screen four complex mixtures extracted and purified from the frontal and occipital lobes (FL, OL) of 20- and 82-year-old male brains. The authors revealed a decrease in the numbers of GG as well as in the degree of sialylation, fucosylation, and acetylation of GGs with aging. They also noted a high variability of sialylation within regions, which correlated with a high diversity of ceramide constitution for certain species. Another study focused on the comparative screening and structural analysis of GGs expressed in fetal and aged cerebellum using Orbitrap MS with nanoelectrospray ionization (nano-ESI) in the negative ion mode (Ica et al., 2020), considering that cerebellar dysfunctions are related to AD (Mavroudis, 2019). They found several GGs, particularly polysialylated ones belonging to the GT, GQ, GP, and GS classes, modified by *O-*fucosylation, *O-a*cetylation, or CH3COO-; these were discovered for the first time in the human cerebellum. These components were differently expressed in fetal and aged tissues, indicating that the GG profile in the cerebellum is development stage- and age-specific, which are attributable to the neurodevelopmental process and may be applied accordingly as markers of cerebellum aging. The sphingosine and fatty acid components of the GGs can induce a change in the developing human brain (Rosenberg and Stern, 1966). The sphingosine portion of the brain GGs changes from almost exclusively C18 at birth to nearly equal quantities of C18 and C20 with organ maturation (Rosenberg and Stern, 1966). Kracun et al. (1991) investigated brain GGs in AD. The study found that all ganglio-series GGs (e.g., GM1, GD1a, GD1b, and GT1b) decreased in regions (temporal and frontal cortex and nucleus basalis of Meynert) involved in the disease pathogenesis. In addition, simple GGs (GN2, GM3) were elevated in the frontal and parietal cortex in AD, which may correlate with accelerated lysosomal degradation of GGs and/or astrogliosis occurring during neuronal death. All of these findings are of major value for correlating GGs with various CNS disorders, including AD, and for investigations related to the development of GG-based therapies.

Sialylation is one form of glycosylation modification (Lee and Wang, 2020). Sialic acids (SA) possess a nine-carbon skeletal structure with a negative charge and high hydrophilicity, making them the most enriched sugar residues found at the terminals of polysaccharides (Liao et al., 2020). There are three forms of SAs: *N-*acetylneuraminic acid (Neu5Ac), *N-*glycolylneuraminic acid (Neu5Gc), and ketodeoxynonulosonic acid. Mixed Neu5Gc and Neu5Ac exist in rats, swine, and tree shrews, while Neu5Ac exists solely in humans due to the lack of cytidine monophosph *O-N-*acetylneuraminic acid hydroxylase (Bern et al., 2013; Walther et al., 2013). SAs have various biological functions, including regulating the immune system, maintaining neural tissues, and influencing cancer malignancy (Wielgat and Braszko, 2012; Schauer and Kamerling, 2018). Abnormal sialylation levels have been associated with cancer, inflammatory diseases, and NDDs (van Kamp et al., 1995; Munkley, 2022). Imbalances in liver metabolic pathways and inflammatory homeostasis can lead to excessive sialylation of liver cells (Lau et al., 2007). Sialylated modifications are essential for maintaining the homeostasis of intestinal mucus, providing necessary nutritional support for intestinal microflora. Desialylation of intestinal mucin damages the intestinal mucosal barrier, contributing to the onset of inflammatory bowel disease (Yao et al., 2022). For instance, in the CNS, activated microglia increase sialidase activity, reducing cell surface sialylation and stimulating microglial phagocytosis of neurons (Rutishauser, 2008). Understanding the interactions between sialylation and disease pathology and elucidating the underlying mechanisms of abnormal glycan sialylation levels in pathological environments can lead to the identification of novel molecular targets for treating complex diseases.

**Figure 2:**
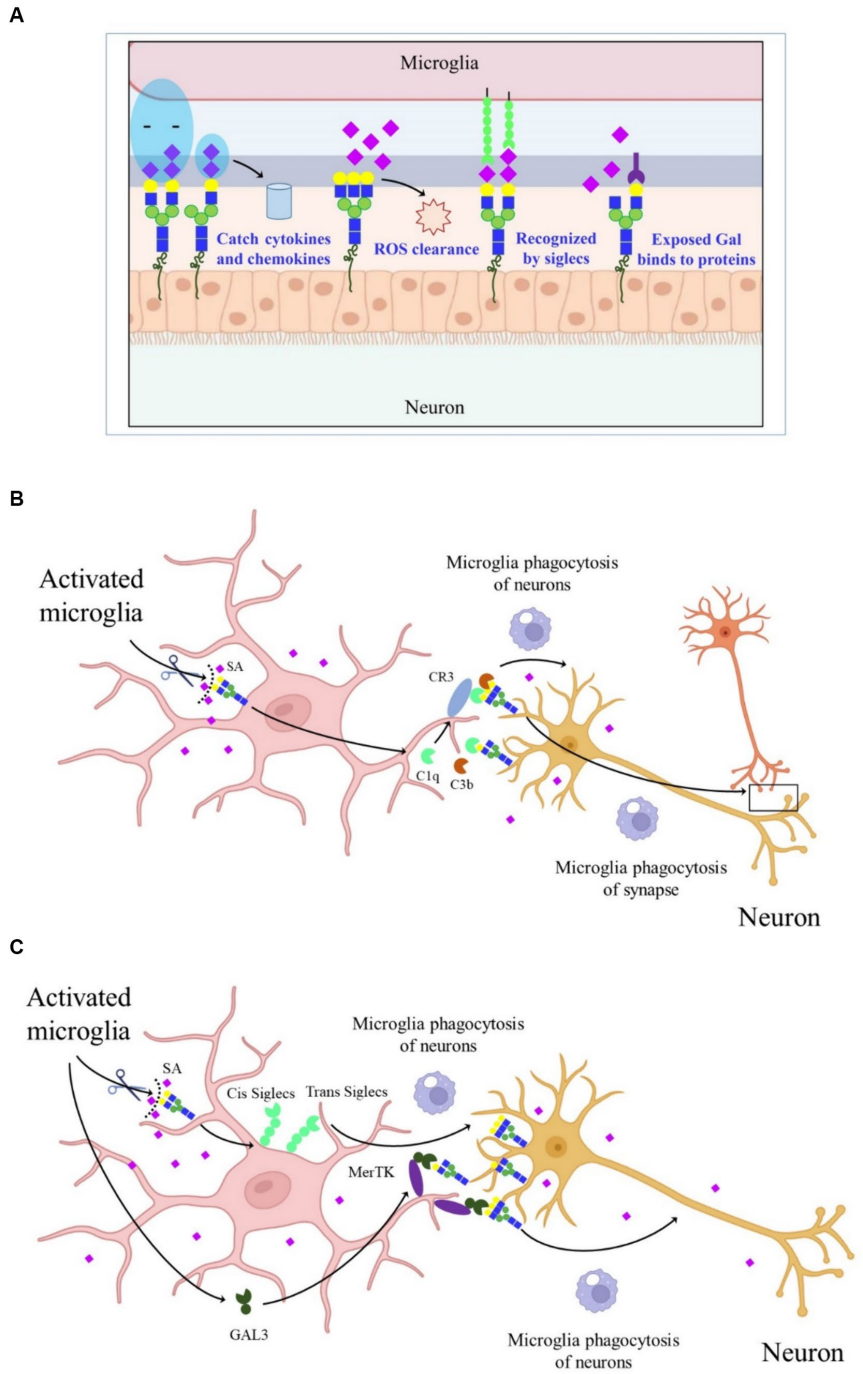
Schematic graph depicting the molecular mechanisms of SAs in CNS. **(A)** The effect of SAs in CNS. **(B)** CR3-dependent phagocytosis of microglia. Activated microglia increased the sialidase activity and desialylated microglia and neurons, making the released C1q and C3b firmly bind to glycans chains on neurons and allow detection by CR3, thereby promoting microglia phagocytosis of synapse and neurons. **(C)** Desialylated microglia and surrounding neurons reduced the binding of *cis*- and *trans-*SIGLECs, released GAL-3, and bound to GAL on the glycan chains, thereby increasing microglia phagocytosis of neurons through MerTK.

There are approximately 20 times more SAs in the human CNS than in other tissues, with the majority present in GGs, highlighting the critical role of SAs in maintaining normal nerve structure and function. SAs play a key role in regulating various processes in the CNS, including synaptogenesis, neurogenesis, cell proliferation and migration, axon guidance, muscle bundle tremor, learning, memory, and cell adhesion. Additionally, the widespread distribution of negatively charged SAs is involved in capturing neurotrophic factors, growth factors, neurotransmitters, ions, cytokines, chemokines, and transcription factors. Sialylated nerve cells can act as receptors or ligands in cell–cell interactions, while free SAs contribute to scavenging reactive oxygen species (ROS) (Figure 2A).

The SAs on glycoconjugates also regulate neuroinflammation (Liao et al., 2020). Activated microglia exhibit increased sialidase activity, leading to decreased sialylation levels, including the enzymes Neu1, Neu2, Neu3, and Neu4 in nerve cells like microglia and astrocytes, particularly under acute stress conditions (Abe et al., 2019). Knockdown of Neu4 in the hippocampus prolongs escape latency in the Morris water maze experiment, indicating that sialidase regulation of SA signaling can affect hippocampal memory processes (Minami et al., 2016). Therefore, neuroinflammation may be related to the transfer of sialidases to the cell surface, leading to changes in sialylation. Mice with reduced SAs display microglia activation and neuronal synaptic loss, combined with complement-dependent neuronal loss, suggesting that SAs are essential for maintaining microglial phagocytosis and normal brain physiological functions (Klaus et al., 2020). Polysialic acids (polySia), enriched in the CNS, contain α2,8-linked Neu5Ac with a degree of polymerization (DP) between 8 and 100 (Lünemann et al., 2021). The negatively charged polySia promotes neuronal regeneration and regulates plasticity and repair-related responses in the brain. PolySia can also be recognized by SA-binding immunoglobulin-type lectins. Abundant SAs present in polySia on neural cell adhesion molecules regulate neurite outgrowth and synaptogenesis (Becker et al., 1996).

In AD, the complement-dependent pathway is abnormally activated, leading to synapse loss (Hong et al., 2016). However, the mechanism by which complements bind to synapses during AD development remains unclear. One explanation is that activated microglia increase sialidase activity, leading to the desialylation of microglia and neurons. This desialylation allows released C1q and C3b to firmly bind to glycan chains on neurons, which are then detected by complement receptor 3 (CR3), promoting microglial phagocytosis (Figure 2B) (Puigdellívol et al., 2020). Another proposed mechanism suggests that under inflammatory conditions, activated and desialylated microglia and surrounding neurons reduce the binding of cis and trans-SA-binding immunoglobulin*-*type lectins. Simultaneously, activated microglia release galecti*N-*3 (GAL-3), which binds to exposed GAL on glycan chains, increasing microglial phagocytosis of neurons through Mer tyrosine kinase (MerTK) (Figure 2C). These findings highlight the importance of sialylation in neuroinflammation and the progression of neurodegenerative diseases (NDDs). However, the reasons behind activated microglia causing cell surface desialylation, the direct mediation of microglial phagocytosis by desialylation, and whether inhibiting sialidase activity would prevent NDDs are still unknown.

## 3 Glycosylation is involved in the pathogenesis and etiology of AD

In the brains of patients with AD, amyloid precursor protein (APP) is initially cleaved by β-site APP-cleaving enzyme 1 (BACE1) to generate a soluble -NH2 terminal fragment and a -COOH terminal fragment (C99). Subsequently, γ-secretase cleaves C99 to produce Aβ peptides, which are known to be neurotoxic and contribute to the progression of AD (Association As, 2016). Due to the inaccurate cleavage position of *γ*-secretase, various lengths of Aβ peptides, including Aβ42, Aβ40, and other oligomers, can be produced. Aβ42 peptides are widely distributed in the cerebrospinal fluid (CSF) of patients with AD and are considered potential biomarkers for the disease. The Aβ42/Aβ40 ratio is more efficient than measuring Aβ42 alone in predicting AD pathology (Baiardi et al., 2019). Current treatment strategies for AD focus on inhibiting BACE1 and γ-secretase activity or increasing Aβ clearance. However, these strategies have shown limited efficacy, and irregular phenotypes, such as schizophrenia and retinal pathology, have been reported in BACE1-deficient mice (Cai et al., 2012). The development of effective drugs has been challenging, and some studies suggest that targeting Aβ may not be the most appropriate approach for managing AD. Therefore, there is an urgent need to expand our understanding of AD pathogenesis to develop complementary or alternative treatment strategies (Schedin-Weiss et al., 2014).

In 2016, the first detailed and systematic report on CSF *N-*glycome profiling in potential patients with AD identified over 90 *N-*glycans, some of which were significantly altered and served as potential biomarkers for early AD diagnosis. This finding strongly suggests that monitoring glycosylation alterations could be a promising and accurate tool for AD detection (Palmigiano et al., 2016). Additionally, *N-*glycome analysis of the cortex and hippocampus from five patients with AD revealed that over 70% of *N-*glycans were complex type *N-*glycans decorated with fucose and SAs, while high-mannose type *N-*glycans accounted for approximately 20% (Gaunitz et al., 2021a). Recently, unbiased, large-scale qualitative and quantitative *N-*glycoproteome profiling using high-resolution mass spectrometry (Zhang et al., 2020) detected 1,333 *N-*glycosylation sites in both patients with AD and control groups. Of these, 698 and 263 *N-*glycosylation sites were characteristic of patients with AD and controls, respectively. Notably, 118 *N-*glycopeptides were significantly altered in patients with AD compared to controls, with 20 specific *N-*glycopeptides/glycosites identified as attractive targets for AD identification (Zhang et al., 2020).

*N-*glycans and *O-*glycans play crucial roles in the function of proteins involved in AD, such as APP, BACE1, and *γ*-secretase. The glycosylation of APP in Chinese hamster ovary cells is characterized by complex, fucosylated, and non-fucosylated *N-*glycans with bi- or tri-antennary structures (Sato et al., 1999). A systematic mapping of *N*-glycoproteins and *N*-glycosylation sites on AD-related proteins (including APP, tau, nicastrin, BACE1) and genes associated with AD was thoroughly performed in genome-wide association studies. The number of *N-*glycosylation sites of these proteins (except tau) was increased in AD patients relative to those in controls because they can reach *N*-glycosylation enzymes (OST complexes) that reside in the ER lumen, which alters the activity of corresponding enzymes. These results together strongly support the role of abnormal *N-*glycosylation in AD pathogenesis (Zhang et al., 2020). These alterations in *N-*glycosylation sites in AD may lead to genetic variations, transcriptional disorders, protein expression imbalances, and disturbances in post-translational modifications. Additionally, a significant correlation between confirmed *N-*glycopeptide/*N-*glycoprotein co-regulatory modules and neurofibrillary tangle pathology, as well as endoplasmic reticulum stress induced by tau deposition, has been proposed (Zhang et al., 2020). Dysregulated *N-*glycosylation in AD brains affects various processes, including synaptic dysfunction, neuroinflammation, lysosomal dysfunction, changes in cell adhesion, endoplasmic reticulum dysfunction, extracellular matrix dysfunction, endocytic transport disorders, and cellular signaling disorders (Zhang et al., 2020).

The APP is reported to have two potential *N-*glycosylation sites, located at Asn467 and Asn496, respectively. Loss of these glycosylation sites leads to reduced secretion and microsomal localization of APP (Table 1) (Yazaki et al., 1996). APP has both high-mannose and complex *N-*glycan structures (Kizuka et al., 2017). Tunicamycin, an *N-*glycosylation inhibitor, can accelerate the mis-sorting of wild-type APP (Tienari et al., 1996). Additionally, some *O-*glycosylation and *O-*GlcNAcylation sites of APP, such as Ser597, Ser606, and Ser662, have been identified in human CSF (Perdivara et al., 2009). Both *N-* and *O-*glycosylated APP can regulate Aβ secretion. Other proteins involved in AD pathology, such as tau and nicastrin (a component of *γ*-secretase), are also *O-*GlcNAcylated. Tau, a phosphoprotein, has 47 potential *N-*glycosylation sites and is responsible for microtubule assembly and disassembly (Gao et al., 2018). High-mannose and complex sialylated *N-*glycans have been identified in paired helical filament-tau, while nicastrin contains a mixture of high-mannose, hybrid, and complex *N-*glycans. Phosphorylated tau has bisecting GlcNAc *N-*glycan structures (Wang et al., 1996; Ciurea et al., 2023). Glycosylated tau has only been observed in the brains of patients with AD, and the reasons for this observation remain unknown (Sato et al., 2001). Patients with AD exhibit low *O-*GlcNAc levels and tau hyperphosphorylation, accompanied by impaired glucose metabolism (Liu et al., 2004). *O-*GlcNAcylation affects APP processing to some degree, but its exact functions are still poorly understood (Griffith et al., 1995).

**Table 1:** Glycosylation of AD-related proteins.

Disease	Protein	Glycosylation sites	Glycosylation type	Functions
AD	APP	2 potential *N-*glycosylation sites—Asn467 and Asn496 *O-*glycosylation sites: Thr291, Thr292, Thr576, Ser597, Ser606, Ser662, Ser611, Ser680, Thr616, Thr634, and Thr635	*O-*GlcNAcylated *N-*glycosylated.	The overexpression of APP *α*2,6-sialylation promotes Aβ secretion *O-*GlcNAcylation of APP inhibits the endocytosis of APP and decreases Aβ secretion
	BACE1	4 potential *N-*glycosylation sites No *O-*glycosylation sites	*N-*glycosylated	Bisecting GlcNAc modifies BACE1, assists BACE1 to escape from lysosomal degradation, and promotes Aβ generation
	Nicastrin	16 potential *N-*glycosylation sites	*O-*GlcNAcylated *N-*glycosylated	Glycosylated nicastrin may affect recognition and interactions with ligands, substrates, and other molecules. Deficiency of *O-*GlcNAcylated nicastrin decreases Aβ generation
	Tau	47 potential *N-*glycosylation sites	*O-*GlcNAcylated *N-*glycosylated	A low level of *O-*GlcNAcylated tau was detected in AD patients’ brains and affected APP processing
	PS	No glycosylation sites	Unknown	Unknown

BACE1 is a type 1 transmembrane protein that forms a subfamily of membrane-anchored aspartate proteases with BACE2 (Yan, 2017). As a rate-limiting enzyme in Aβ generation, BACE1 undergoes post-translational or co-translational modifications, including *N-*glycosylation. BACE1 has four potential *N-*glycosylation sites and no *O-*glycosylation site (Gaunitz et al., 2021b). Tomita et al. (1998) revealed that the activity of BACE1 was affected by its glycosylation pattern, and, after APP was modified by *O*-glycosylation in the Golgi apparatus, the cleavage of APP by *α*-secretase, *β*-secretase, and *γ*-secretase begins, indicating that the pathways by which APP is processed and hydrolyzed is influenced by its glycosylation pattern. Moreover, further research proved that different APP cleavage patterns have distinct lectin-binding patterns that determine the processing and hydrolysis modes of APP (Boix et al., 2020). In addition to the sequential cleavage of APP, *β*-secretase and *γ*-secretase can affect the glycosylation and complex *N-*glycosylation of APP by other mechanisms; these processes play important roles in AD pathogenesis, implying the numerous worthwhile inter-regulatory relationships between APP and various secretory enzymes that need to be further explored (Schedin-Weiss et al., 2014). Current strategies regarding the inhibition of APP processing by altering glycosylation have focused on the regulation of hydrolases, such as the curcumin derivative GT863. In addition, considering that previous inhibitors designed for BACE1 have been reported to generate serious side effects, a selective modulation of the cleavage activity of BACE1 by altering the glycosylation pattern (such as modulating galactosyltransferases and mannosidases) to regulate the effects of glycosylases is being considered as a promising and novel therapeutic modality for AD (Nahálková, 2022).

Among the four protein components of *γ*-secretase, only nicastrin is glycosylated and has 16 potential *N-*glycosylation sites. The physiological effects of glycosylated nicastrin may affect recognition and interactions with ligands, substrates, and other molecules, but other physiological roles need further exploration. Deficiency of *O-*GlcNAcylated nicastrin decreases Aβ generation (Moniruzzaman et al., 2018; Gaunitz et al., 2021b). The complex glycosylation of nicastrin is not essential for *γ*-secretase activity but depends on presenilin (PS), a transmembrane protein that forms a complex with APP and participates in the transport and post-synthesis processing of APP (Herreman et al., 2003). A significant decrease in nicastrin was observed in cells lacking PS1, while a slight decline in the amount of nicastrin was found in PS2-deficient cells (Herreman et al., 2003). Additionally, altered glycosylation of acetylcholinesterase and mannan-binding lectin has been observed in the frontal cortex, serum, and CSF of severe AD cases (Saez-Valero et al., 2000). Mannan*-*binding lectin can activate the classical complement pathway, and proteins in this signaling pathway have been found in patients with AD; these alterations typically occur in the late stages of AD progression and are not suitable as biomarkers for early AD diagnosis. PS1 reportedly affects the *N-*glycosylation process directly or indirectly, thereby altering the subcellular location of proteins; this effect on glycosylation alters the subcellular distribution and turnover of telencephalin, APP, and APP-like protein 1 (Annaert et al., 2001), and the underlying mechanisms remain to be clarified.

Therefore, considering the other physiological functions of AD-related proteins and the importance of protein glycosylation in AD progression, an in-depth investigation of altered glycosylation of AD-related proteins instead of directly targeting them may be safer and could provide novel ideas for the diagnosis, treatment, and prevention of AD.

## 4 Sialyltransferases and AD

**Figure 3:**
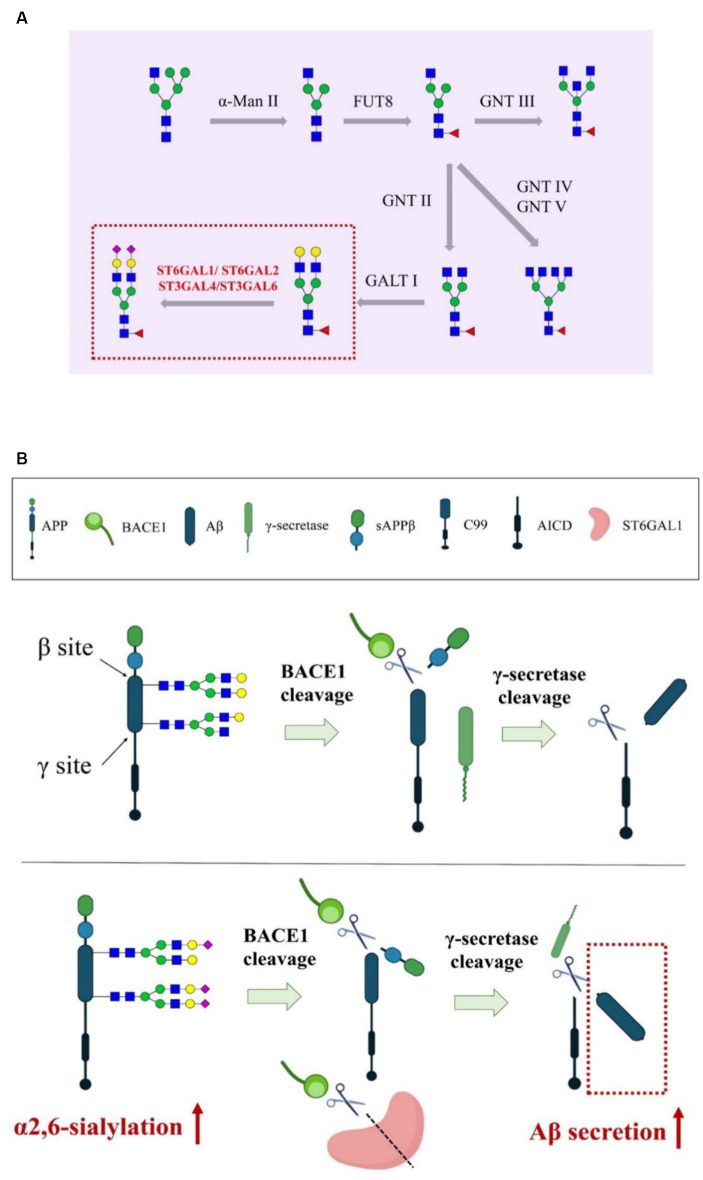
The model of Aβ secretion regulated by STs. **(A)** The role of STs in mammalian *N-*glycosylation modification. **(B)** The overexpression of APP *α*2,6-sialylation driven by ST6GAL1 promoted BACE1-led cleavage of ST6GAL1 and APP, resulting in the accumulation of Aβ peptides and acceleration of AD progression.

*α*2,3- and *α*2,6-linkages are two major linkages between SAs and the penultimate galactose residue, formed by sialyltransferases (STs). The human genome encodes over 20 different STs, all of which use cytidine monophosphate *N-*acetylneuraminic acid (CMP-Neu5Ac) as activated sugar donors to transfer SAs to the terminal of glycoproteins or glycolipids. The ST family is divided into four major groups based on the linkage type: GAL *α*2,3-STs, GAL *α*2,6-STs, GALNAc *α*2,6-STs, and *α*2,8-STs GAL *α*2,6-STs and GALNAc *α*2,6-STs are responsible for synthesizing the *α*2,6 configuration, while GAL *α*2,3-STs are responsible for synthesizing the *α*2,3 configuration in mammalian cells (Figure 3A).

ST6GAL1 and ST3GAL4 are highly expressed in human respiratory tract tissues. Their abnormal expression levels lead to an imbalance in cell sialylation, affecting the process of disease development by regulating the transcriptional activity of HIF-1α, Spil, and NF-κB, among others. This imbalance promotes the development of inflammation or the transformation of inflammation and cancer, making them potential targets for the prevention and treatment of cancer and several inflammatory diseases. The mutation of the GG-specific ST gene ST3GAL5 leads to severe infantile seizures, developmental retardation, and blindness (Boccuto et al., 2014). ST3GAL3 mutation is characterized by intellectual disorder and infantile epilepsy (Edvardson et al., 2013); a deficiency of ST3GAL2 results in dysmyelination characterized by a dramatic decline in myelin proteins and myelin thickness (Yoo et al., 2015).

In the CNS, the polymorphism of ST6GAL1 is related to the deterioration of mild cognitive impairment to AD (Lee et al., 2017). The activity of STs responsible for *α*2,6- and *α*2,3-sialylation is significantly decreased in patients with AD compared to healthy individuals, and the degree of CSF protein sialylation in patients with AD is remarkably lower than that in those without AD (Gaunitz et al., 2021b). Loss of sialylation is a signal of protein aging and can lead to the removal of synapses and neurons, thus, sialylation and STs act as a checkpoint in the CNS (Klaus et al., 2021). In fact, ST6GAL1 is one of the substrates of BACE1, and several reports have demonstrated that BACE1-driven ST6GAL1 processing is necessary for the production of a soluble form of STs (Kitazume et al., 2001). The plasma ST6GAL1 levels in BACE1-knockout mice are only one-third of those in control mice. ST6GAL1 is markedly downregulated when co-expressed with APP, and its secretion significantly increases in BACE1-overexpressed cells, indicating that BACE1 is not only responsible for the cleavage and secretion of APP but also ST6GAL1 (Figure 3B); APP cleavage competes with ST6GAL1 processing (Ciurea et al., 2023). Co*-*overexpression of BACE1 and ST6GAL1 increases the degree of soluble secretory glycoprotein rather than cell surface sialylation (Sato et al., 2001). Interestingly, a high level of APP sialylation driven by overexpressed ST6GAL1 promotes APP secretion, leading to a 2-fold increase in Aβ, 3-fold increase in soluble APPβ (sAPPβ), and 2.5-fold increase in sAPPα in the extracellular level of its metabolites; sialylation-deficient mutant CHO cells secreted half the amount of Aβ as the wild-type cells (Nakagawa et al., 2006). These results together suggest that the cleavage of ST6GAL1 by BACE1 promotes APP metabolic turnover and can regulate the pathological development of AD (Nakagawa et al., 2006). Although BACE-1 affects the glycosylation of secreted proteins, it does not have any effect on the glycoproteins on the cell surface, possibly because soluble ST6GAL1 possesses the ability to move more freely due to the loss of its membrane-anchoring region, which may improve the catalytic response of soluble glycoproteins in the trans-Golgi network or secretory vesicles (Sugimoto et al., 2007). In response to α2,3-sialylation, although the altered cleavage of ST3GAL1, ST3GAL2, ST3GAL3, and ST3GAL4 could not be detected *in vitro*, the overexpression of BACE-1 in COS cells enhanced the secretion of these substances; one of the explanations for this observation is that BACE-1 can activate several proteases responsible for the processing of ST3GAL family or that BACE-1 inactivates the retention mechanism of ST3GAL proteins in the Golgi apparatus (Kitazume et al., 2006). Presently, other studies have also confirmed that Tyr10 glycosylated Aβ peptides were remarkably increased in the CSF of AD patients, which implies that sialylated *O*-glycans influence APP processing (Halim et al., 2011).

Unlike ST6GAL1, which is commonly expressed in all tissues, ST6GAL2 was only detected in the brain and embryonic tissues, and its expression level was much lower than that of ST6GAL1 (Ohmi et al., 2021). While the ST6GAL1 gene was detected in different types of nerve cells, including microglia, astrocytes, and neurons, ST6GAL2 was primarily found in astrocytes and neurons (Ohmi et al., 2021). ST6GAL2 was the last member of the ST family to be discovered due to its low enzyme activity. It appears to exhibit narrower enzyme substrate specificity compared to ST6GAL1 (Makarava et al., 2022). Although it has been reported that ST6GAL2 can respond to a proinflammatory environment in the brain, its physiological function remains largely unknown (Lehoux et al., 2010).

One of the neurodevelopment-related genes, polysialyltransferase ST8SIA2, is responsible for the synthesis of polySia. The loss of ST8SIA2 has been associated with the occurrence of several major psychiatric conditions (McAuley et al., 2012). Functional impairment of ST8SIA2 affects disease progression in some mental disorders, such as schizophrenia-like behavioral abnormalities (Isomura et al., 2011). Since the etiopathogenesis of AD involves neurodevelopmental processes, ST8SIA2 variants have been associated with the occurrence of mental illness and inflammatory responses. ST8SIA2 may regulate specific symptoms of AD by modulating inflammatory systems (Stefano et al., 2016).

## 5 GnT-III and AD

**Figure 4:**
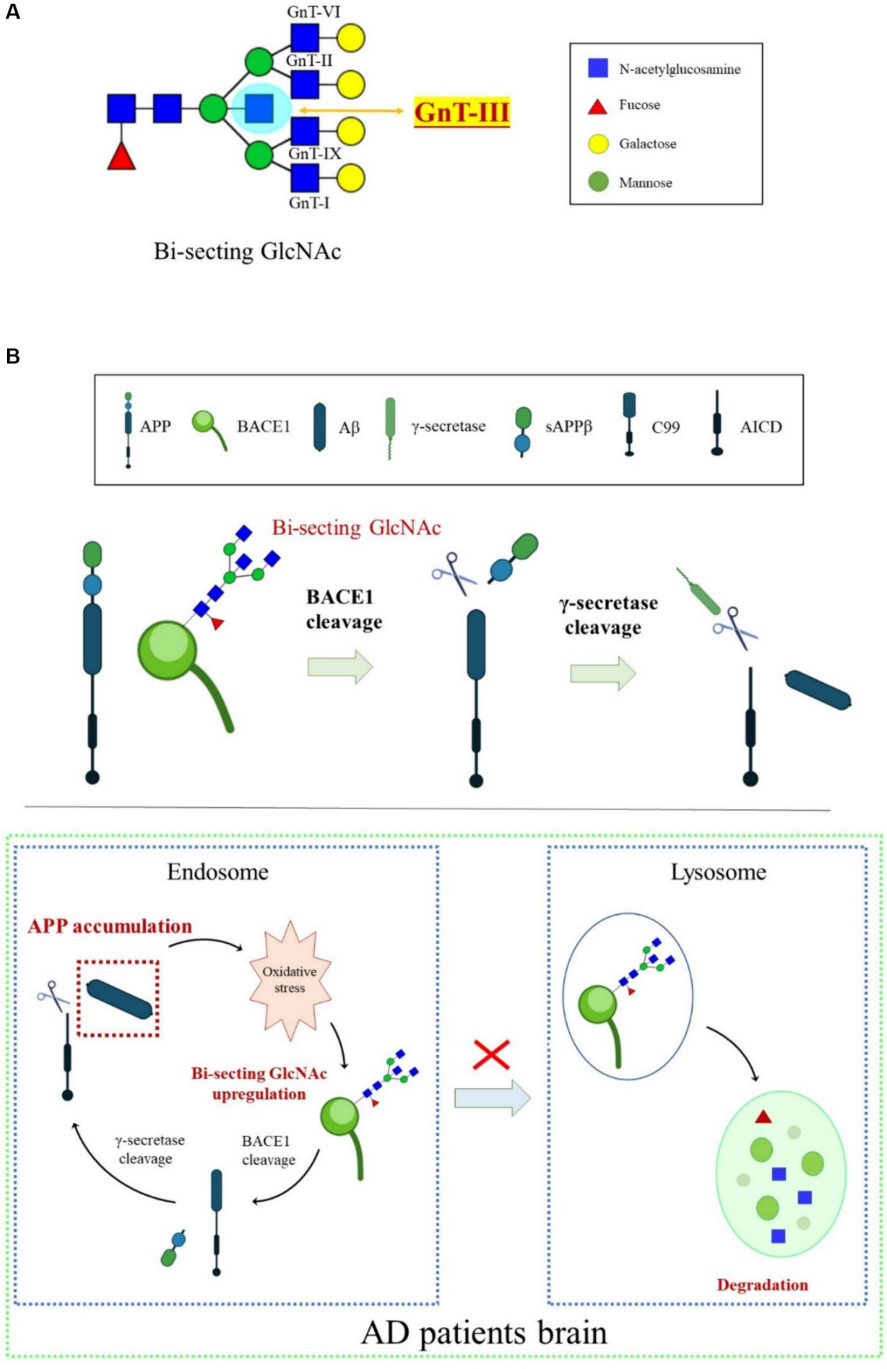
An overview of BACE1 transport regulated by bisecting GlcNAc modification. **(A)** *N-*glycan core modification by different glycosyltransferases. GnT-III is the sole enzyme responsible for synthesizing bisecting GlcNAc. **(B)** Oxidative stress-induced increase in the bisecting GlcNAc levels was involved in BACE1 upregulation by protecting BACE1 from lysosomal degradation, which, in turn, accelerated Aβ generation.

Bisecting GlcNAc is a special type of *N-*glycan synthesized by *N-*acetylglucosaminyltransferase-III (GnT-III) and is highly expressed in the CNS (Figure 4A). Unlike other GlcNAc branches in *N-*glycans, bisecting GlcNAc cannot be further elongated by adding other sugar residues, and the presence of GnT-III affects the activity of other branching enzymes (Schachter, 1986). Overexpression of GnT-III inhibits tumor invasion and metastasis in mice (Yoshimura et al., 1995), while GnT-III-deficient mice decrease the viability of liver cancer cells (Song et al., 2010).

The high expression of GnT-III detected in the temporal cortex of the brains of patients with AD indicates that GnT-III and its synthesized bisecting GlcNAc play an important role in the process of AD, and their abnormal expression is considered a sign of early onset of AD (Kizuka et al., 2015). Glycomic analysis revealed that BACE1 is modified by bisecting GlcNAc in the brains of patients with AD. Loss of GnT-III significantly impairs the cleavage activity of APP by BACE1, contributing to Aβ clearance and remodeling of cognitive function (Kizuka et al., 2015). Further research showed that deficiency of bisecting GlcNAc modification induces early entry of BACE1 into late endosomes/lysosomes, where lysosomal degradation of BACE1 occurs, resulting in less co-localization with APP. However, other BACE1 substrates, such as CHL1 and contact protein-2, are normally cleaved in GnT-III-deficient mice, indicating that bisecting GlcNAc-modified BACE1 selectively cleaves APP (Kizuka et al., 2015). The mechanisms underlying the high level of GnT-III in patients with AD might be related to oxidative stress. Oxidative stress in the brain is toxic to cell growth, and over-generated ROS can promote the activity and transcription of BACE1. Oxidative damage found in AD model mice induces the production of BACE1 protein and bisecting GlcNAc (Chami and Checler, 2012). BACE1 is the targeted protein of GnT-III, and the BACE1 level triggered by oxidative stress in GnT-III-deficient cells was lower. Furthermore, BACE1 degradation in lysosomes appears to be faster in GnT-III-deficient cells than in normal cells, suggesting that the increase in bisecting GlcNAc levels induced by oxidative stress is involved in BACE1 upregulation by protecting it from lysosomal degradation (Takahashi et al., 2016). Lack of GnT-III induces altered positioning of BACE1 from early endosomes to lysosomes. Bisecting GlcNAc is a hallmark for modified protein transport to the endosomal compartment, and an advanced high-throughput screening method has been developed to seek GnT-III inhibitors (Kizuka and Taniguchi, 2018). We proposed that bisecting GlcNAc plays a vital role in a vicious cycle in which Aβ accumulation in patients with AD causes excessive ROS. Oxidative stress upregulates bisecting GlcNAc-modified BACE1, helping BACE1 escape from lysosomal degradation and eventually leading to more Aβ generation in the brain (Figure 4B). Currently, there are no effective GnT-III inhibitors available clinically due to the lack of high-throughput measurement systems to detect GnT-III enzyme structure and activity. However, several small molecular inhibitors, such as microRNA-23b (miR-23b), which targets GnT-III, efficiently interrupt AD pathogenesis by restraining oxidative stress and inhibiting tau-lesion (Pan et al., 2021). Glucagon-like peptide-1 (GLP-1) and its mimetics significantly suppress GnT-III by regulating the Akt/GSK-3β/β-catenin signaling pathway in neurons (Kim et al., 2015). Targeting GnT-III appears to be a safe and promising strategy for AD therapeutics, given that GnT-III deficiency shows a slight phenotype and does not cause fatal damage *in vivo*. Further clarification is needed on how GnT-III is affected by oxidative stress and which other proteins are targets of GnT-III.

## 6 Fucosyltransferase and AD

Compared with healthy individuals, four glycans in the hippocampus and two glycans in the cortex show marked expression changes in AD. Besides the bisecting GlcNAc and SA-linked structures mentioned above, all of these glycans possess a core fucose configuration, suggesting that fucosyltransferases (FUT) might be involved in AD pathogenesis (Gaunitz et al., 2021a). Deactivation of antioxidant enzymes induced oxidative stress, which is proposed to be an important factor promoting Aβ deposition and contributing to AD progression. GlycoMaple is a novel technology that helps profile glycan structures based on the detection of glycan biosynthesis-related enzymes. Glycans with a core fucose structure and its synthetase FUT8 are upregulated after the antioxidant response. This is mainly due to its location and activity alteration after a change in redox status. Therefore, core fucose may serve as a novel biomarker for oxidative stress. Upstream transcription factors of FUT8 upon oxidative stress are poorly understood. The upregulation of FUT8 expression may be independent of the antioxidative response, and the resulting increase in core fucose may be protective for cells under excessive ROS conditions (Kyunai et al., 2023). However, FUT8-deficient mice exhibit serious delayed growth and postpartum death (Taniguchi et al., 2022).

Aβ alters the expression of FUT9, which is distributed in neural stem cells and is responsible for Lewis X carbohydrate epitope synthesis. Aβ can promote neural stem cell proliferation by upregulating FUT9, which is involved in classical Notch signaling (Itokazu and Yu, 2014). Contrary to the above research, bi-antennary SA-linked glycans lacking core fucose were overexpressed in the CSF of patients with AD using MALDI-TOF mass spectrometry (Florentinus-Mefailoski et al., 2021). Little is known about the role and significance of core fucose and FUT in the brains of patients with AD and whether regulating their expression can affect the progression of AD, which warrants further investigation.

## 7 *O-*GlcNAcylation and AD

**Figure 5:**
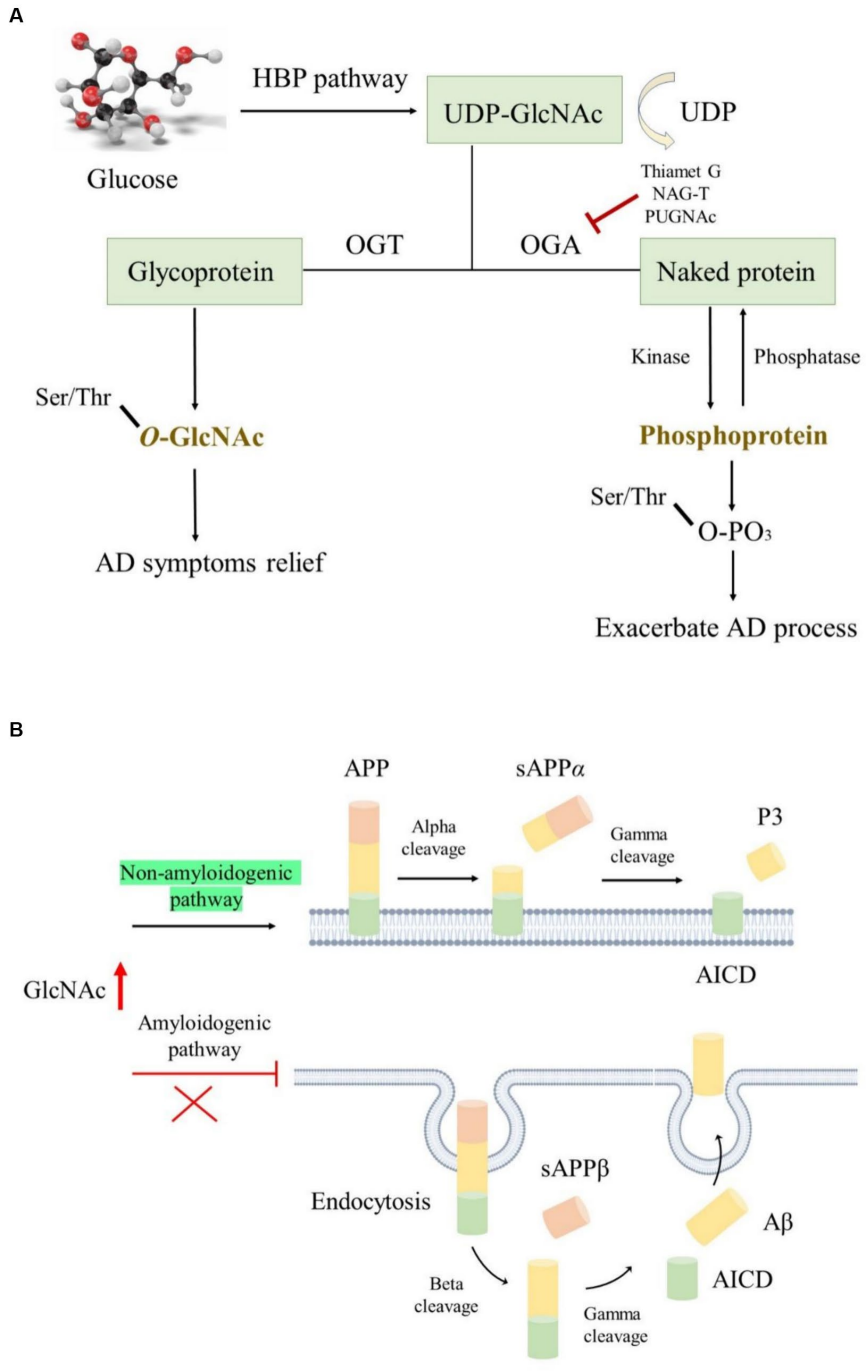
The roles of *O-*GlcNAcylation modification in AD. **(A)** *O-*GlcNAcylation utilizes UDP-GlcNAc as donors produced by the HBP pathway; OGA and OGT regulated the *O-*GlcNAc level, while kinase and phosphatase modulated the protein phosphorylation level; elevated *O-*GlcNAc level is conducive to ameliorating AD symptoms. **(B)** Increased *O-*GlcNAcylation activated the non-amyloidogenic pathway and facilitated endocytosis evasion of APP, thereby reducing Aβ generation.

Opposite to the synthesis of *N-* or *O-*linked glycosylation, which involves several enzymes, *O-*GlcNAcylation is a unique process occurring in the brain. It is catalyzed positively by glycosyltransferases like *O-*GlcNAc transferase (OGT) and epidermal growth factor domain-specific *O-*GlcNAc transferase while being negatively regulated by *O-*linked β-*N-*acetylglucosaminidase (OGA). Interestingly, the highest expression of OGT and OGA is found in the human pancreas and brain, indicating their critical roles in these organs (Okuyama and Marshall, 2003; Bukke et al., 2020). This catalytic interplay maintains a dynamic equilibrium, where preserving optimal *O-*GlcNAc levels protects tissues and cells from damage, thereby reducing stress-induced apoptosis (Park et al., 2020). Glucose is the main energy source for the maintenance of normal physiological functions of the adult brains; it is transported from the periphery via the blood–brain barrier to the hippocampus and cortex, where it is utilized for ATP generation to meet the demands of life (Tang, 2020). *O-*GlcNAcylation primarily relies on uridine diphosphate *N-*acetylglucosamine (UDP-GlcNAc) as a donor and is closely associated with glucose availability (Hardivillé and Hart, 2014). This crucial substrate, derived from the hexosamine biosynthetic pathway, is finely tuned by glucose concentration (Figure 5A). Hyperglycemic conditions often elevate *O-*GlcNAc levels, while decreased glucose concentrations lead to a decline in *O-*GlcNAc levels (Clark et al., 2003; Li et al., 2006).

The pathophysiology of various NDDs, including Parkinson’s disease, Huntington’s disease, and AD, has been closely associated with nerve cell aging and impaired glucose metabolism (Park et al., 2020). Notably, alterations in glucose metabolism often precede pathological changes in AD, further exacerbating Aβ accumulation and abnormal tau hyperphosphorylation (An et al., 2018). In AD, decreased expression of glucose transporters such as GLUT1 and GLUT3 results in low glucose levels and diminished *O-*GlcNAcylation (Pinho et al., 2019). Paradoxically, studies have reported elevated *O-*GlcNAc levels in the brains of patients with AD, indicating complex regulatory mechanisms (Förster et al., 2014). Moreover, proteomic analyses have identified several proteins with reduced *O-*GlcNAcylation levels in AD brains, implicating their involvement in key pathways of AD progression (Tramutola et al., 2018). This highlights the intricate interplay between *O-*GlcNAcylation and AD pathology, suggesting the need for comprehensive analyses across different brain regions to elucidate their relationship further.

The APP and tau undergo *O-*GlcNAcylation modifications in AD, and increasing *O-*GlcNAc levels ameliorate AD symptoms. However, the underlying mechanisms are not yet fully understood (Park et al., 2020). *O-*GlcNAcylated APP is thought to activate the non-amyloidogenic pathway instead of the amyloidogenic pathway by suppressing the basic stages of endocytosis. This inhibition of endocytosis reduces Aβ secretion (Chun et al., 2015). Mutation of *O-*GlcNAcylation sites on APP can lead to its escape from endocytosis, further decreasing Aβ generation (Figure 5B). Interestingly, Aβ generation can also affect *O-*GlcNAc levels, as long-term exposure to Aβ reduces OGT activity, resulting in an overall reduction in *O-*GlcNAc levels (Ryu et al., 2016). Moreover, some phosphorylated tau residues undergo *O-*GlcNAcylation modifications, which inhibit tau accumulation by reducing its phosphorylated form (Acosta-Baena et al., 2011), thereby improving AD symptoms. *O-*GlcNAcylated tau levels are negatively correlated with phosphorylated tau levels in patients with AD due to impaired glucose metabolism (Lefebvre et al., 2003; Liu et al., 2004). In fasting mice, decreasing glucose levels are accompanied by a decrease in *O-*GlcNAcylated tau and an increase in phosphorylated tau, indicating a delicate balance between these two modifications (Lefebvre et al., 2003). Several reports suggest that using OGA inhibitors such as Thiamet G, NAG-T, or PUGNAc is a promising treatment approach for tau-related NDDs. Long-term treatment with Thiamet G in mice resulted in a remarkable decrease in several tau protein abnormalities (Hastings et al., 2017), although no significant effects were observed following 4 weeks of treatment. Additionally, alterations in *O-*GlcNAcylation affect *β*-amyloid protein pathology in the presence of tau (Gatta et al., 2016).

Necroptosis and programmed necrosis have been reported to be closely related to neuronal death or neuroinflammation associated with AD (Caccamo et al., 2017). Inhibiting necroptosis can efficiently alleviate neuroinflammation. Reduced *O-*GlcNAcylation levels in mouse brains induce inflammatory responses and neurodegeneration (Wang et al., 2016). Insufficient expression of OGA, responsible for removing *O-*GlcNAc from proteins, suppressed necroptosis in mouse brains, resulting in reduced Aβ accumulation, cognitive recovery, decreased neuroinflammation, and normal mitochondrial function (Park et al., 2021). This suppression occurred through *O-*GlcNAcylation of receptor-interacting serine/threonine protein kinase 3 (RIPK3), which blocked its self-phosphorylation and the interaction between RIPK1 and RIPK3, thereby transforming proinflammatory microglia (M1) into an anti-inflammatory phenotype (M2). These findings suggest that *O-*GlcNAcylation may serve as a major mediator of RIPK3 to hinder necroptosis and ameliorate AD pathology, offering a novel treatment approach for AD.

The brains of patients with AD exhibit a global decrease in *O-*GlcNAcylation, which is closely related to impaired mitochondrial bioenergetic function, mitochondrial network, and cell viability. Restoring *O-*GlcNAcylation levels reactivates cell viability and physiological functions, suggesting that *O-*GlcNAcylation may serve as a potential link between mitochondrial energy crisis and synaptic and neuronal degeneration in AD pathology (Schachter, 1986). Additionally, *O-*GlcNAcylation has been reported to affect mitochondrial transport by targeting the movement regulator Milton, which is responsible for binding mitochondria to motor proteins (Schachter, 1986). Overall, targeting *O-*GlcNAcylation represents a promising therapeutic option for AD.

## 8 Glycation in AD

Reducing sugars can react non-enzymatically with lysine or arginine side chain amino groups of proteins and form advanced glycation end-products (AGEs), this complex reaction is called the Maillard reaction (Reddy et al., 2022). In the first stage of AGE synthesis, a non-enzymatic condensation reaction occurs between the α-amino or N-terminal group of a protein, lipid, or nucleic acid and the carbonyl group of a reducing sugar (Forbes et al., 2003). Schiff base is formed by a highly reversible nucleophilic addition reaction, followed by a very slow chemical rearrangement of Schiff base, resulting in reversible ketoamine synthesis. Finally, protein aggregates or AGEs are formed after dehydration and rearrangements of this ketoamine (Baynes and Thorpe, 1999; Chellan and Nagaraj, 2001).

The formation of AGEs occurs mainly in the presence of hyperglycemia, hyperlipidemia, and oxidative stress conditions. For instance, AGEs can induce oxidative stress, which increases the intracellular free radical production and incurs damage to cell membranes and organelles; AGEs can activate NF-κB, initiate inflammatory response pathways, and promote the release of inflammatory factors, leading to the persistence and exacerbation of inflammatory responses. AGEs can also induce apoptosis, which increases cell death and thereby affects the functions of tissues and organs. AGEs can cross-link with proteins in the extracellular matrix, affect the protein structure and function, induce stiffness, and impair the functions of tissues (Reddy et al., 2022).

The degree of AGE accumulation is usually closely correlated with the related disease progression, such as diabetes, cardiovascular diseases, and NDDs. In diabetes mellitus and AD, the rate of AGE formation is accelerated; consequently, they have been considered as potentially useful biomarkers for monitoring the treatment of NDD. Glycation induces the formation of *β*-amyloid protein, *α*-synuclein, transthyretin, copper-zinc superoxide dismutase 1 (Cu, Zn-SOD-1), and prion protein into β-sheet structure—this structural aggregation can cause AD, Parkinson’s disease, amyotrophic lateral sclerosis, familial amyloid polyneuropathy, and prion disease, respectively (Horie et al., 1997; Castellani et al., 2001). AGEs usually exist in *β*-amyloid protein and NFTs (Wong et al., 2001), and it has been reported that plaque portions of AD brains contain higher levels of AGEs compared to age-matched control samples and they actively participate in the progression of AD (Vitek et al., 1994).

In AD brains, the receptors for AGEs (RAGE) are highly expressed on neurons, the attachment of AGEs and RAGE initiate downstream NF-κB, SAPK/JNK/p38 signaling pathways, and induces neuronal cell death (Teismann et al., 2012). Diabetes mellitus may be closely related to AD occurrence considering that there are more AGEs and RAGE deposits in the brain of patients with diabetes, which may induce neuroinflammation through the abovementioned signaling pathway (Coker and Wagenknecht, 2011). Nowadays, the AGE-RAGE axis regulation has been considered a promising treatment option for AD.

Li et al. (2013) reported that Aβ is one of the substrates for glycation and produces AGEs, and the formation of Aβ-AGE may exacerbate neurotoxicity by increasing RAGE and glycogen synthase kinase-3 (GSK-3). Meanwhile, the concomitant application of RAGE antibodies or GSK-3 inhibitors reversed the neuronal damage exacerbated by glycated Aβ. Furthermore, a subcutaneous injection of aminoguanidine to inhibit Aβ-AGE could significantly alleviate early cognitive deficits in mice, for example, Li et al. (2013) revealed that glycated Aβ is more toxic and that glycated Aβ may be a promising target for AD treatment for the first time. Similarly, glyceraldehyde, the AGE-derived product, was detected in the hippocampus and parahippocampal gyrus, where it triggers carbonyl stress by suppressing the catalytic activity of GAPDH, leading to the accumulation of glyceraldehyde and methylglyoxal in the brain and the occurrence of AD, which indicates that glycation plays a vital role in AD progression (Butterfield et al., 2010).

## 9 Conclusion

In summary, we have presented evidence of the molecular mechanisms of glycosylation, including sialylation, bisecting GlcNAc, fucosylation, and *O-*GlcNAcylation, in the pathogenesis and etiology of AD. The alteration of protein glycosylation may pave the way for novel therapeutic strategies for AD. However, the underlying mechanisms of glycosylation in AD remain unclear, underscoring the need for further exploration to advance the development of new treatment strategies for AD.

## Author contributions

YK: Conceptualization, Investigation, Writing – original draft. QZ: Data curation, Formal analysis, Resources, Visualization, Writing – original draft. SX: Conceptualization, Methodology, Project administration, Writing – review & editing. YY: Supervision, Validation, Writing – review & editing.

## Glossary

AD: Alzheimer’s disease
APP: Amyloid precursor protein
Aβ: Amyloid-β
AChE: Acetylcholinesterase
BACE1: β-site APP-cleaving enzyme 1
CSF: Cerebrospinal fluid
CNS: Central nervous system
CR3: Complement receptor 3
ER: Endoplasmic reticulum
EOGT: Epidermal growth factor domain-specific O-GlcNAc transferase
FUT: Fucosyltransferases
GAL: Galactose
GWAS: Genome-wide association studies
GLUT: Glucose transporter
GPI: Glycosylphosphatidylinositol
HBP: Hexosamine biosynthetic pathway
MerTK: Mer tyrosine kinase
GALNAc: *N-*acetylgalactosamine
GnT-III: *N-*acetylglucosaminyltransferase-III
Neu5Ac: *N-*acetylneuraminic acid
CMAH: *N-*acetylneuraminic acid hydroxylase
NCAM: Neural cell adhesion molecule
NDD: Neurodegenerative disease
NFTs: Neurofibrillary tangles
NDD: Neurodegenerative disorder
Neu5Gc: *N-*glycolylneuraminic acid
NSC: Neural stem cells
OGA: *O-*linked *β*-*N*-acetylglucosaminidase
OGT: *O-*GlcNAc transferase
P-tau: Phosphorylated tau
DP: Polymerization
PolySia: Polysialic acids
PS: Presenilin
RIPK3: Receptor-interacting serine/threonine protein kinase 3
SA: Sialic acid
SIGLECs: Sialic acid-binding immunoglobulin-like receptors
ST: Sialyltransferase
UDP-GlcNAc: Uridine diphosphate *N-*acetylglucosamine
